# My Heart Has Always Been Set on Pediatrics, and Other Insights From First-Year Medical Students

**DOI:** 10.1093/geroni/igaa057.002

**Published:** 2020-12-16

**Authors:** Jennifer Inker, Sarah Marrs, Madeline McIntyre, Leland Waters, Tracey Gendron

**Affiliations:** Virginia Commonwealth University, Richmond, Virginia, United States

## Abstract

Senior mentoring programs provide medical students exposure to a community-dwelling older adult mentor with whom they meet multiple times throughout the program. The goal of these programs is to expose students to healthy older adults, increase their knowledge of topics in geriatrics and aging, and increase the likelihood that students will pursue geriatric specialties. Though research findings show that senior mentoring programs have the potential to increase medical students’ attitudes towards older adults (Samra et al., 2013) and their willingness to consider working with older patients in the future (McManus et al., 2017), a critical shortage of doctors who specialize in geriatrics still exists. Moreover, the demand for geriatrically-trained physicians is expected to continue to increase (American Geriatrics Society, 2018). In order to develop avenues for successfully fostering interest in and pursuit of geriatrics specialties, we need to fully understand students’ perceptions of working with older adults. The purpose of this qualitative content analysis was to explore first-year medical students’ (n = 216) perceptions of working with older adults. We asked students to respond to the following reflection at the beginning and end of their Senior Mentoring program: How do you feel about working with older patients after you complete your training? Our findings suggest that while students feel more comfortable with and open to caring for older adults, they do not feel compelled to pursue geriatrics. Themes and sub-categories emerging from the data provide insight into why students continue to dismiss pursuing geriatrics.

